# Effect of body inclination on suprahyoid muscle activity during repetitive voluntary swallowing: A randomized crossover study

**DOI:** 10.14814/phy2.70426

**Published:** 2025-06-17

**Authors:** Yumiko Kubo, Hideyuki Fukami

**Affiliations:** ^1^ Department of Oral Health Sciences Faculty of Nursing and Health Care, Baika Women's University Osaka Ibaraki Japan

**Keywords:** body posture, electromyography, pharyngeal region, swallowing

## Abstract

This study examined how body inclination affects swallowing dynamics by analyzing suprahyoid muscle activity during repetitive voluntary swallowing with minimal pharyngeal sensory stimulation. Fourteen healthy female volunteers participated. Surface electromyography (EMG) was recorded from suprahyoid muscles during repetitive voluntary swallowing with slow infusion (0.2 mL/min) of 0.3 M NaCl solution. Measurements were taken at four angles: upright (90°), reclined at 60°, reclined at 30°, and supine (0°). Body inclination significantly affected swallowing intervals (SI) and EMG burst duration. The upright position showed significantly shorter SIs compared to 60° reclined and supine positions. The 30° reclined position also demonstrated shorter swallowing intervals than supine. EMG burst duration was significantly shorter in the upright position compared to all reclined positions. No significant differences were observed in EMG amplitude across different angles. This study demonstrates that greater reclining angles (60° or more) make voluntary repetitive swallowing more difficult. Findings suggest that dysphagia rehabilitation should be conducted at positions elevated at least 30° from supine for optimal outcomes.

## 1 INTRODUCTION

In an increasingly aging society, dysphagia emerges as a significant clinical and societal concern. This condition not only diminishes patients' quality of life by precipitating malnutrition and dehydration but also poses the potential threat of aspiration pneumonia, which can be fatal. The etiology of dysphagia is multifaceted, necessitating diverse rehabilitative strategies (Groher & Crary, 2020). Postural adjustment techniques have been employed to ameliorate anatomical and physiological deficiencies in dysphagic individuals. Inclined body postures are advocated as optimal for facilitating swallowing in patients with dysphagia (Groher & Crary, 2020). The effects of body inclination on swallowing have been studied using various methods.

Numerous investigations have scrutinized the impact of body inclination on swallowing, employing surface electromyography (EMG) to explore the nuanced dynamics (Ayuse et al., 2006; Inagaki et al., 2007, 2008, 2009b; Lund et al., 1970; Miralles et al., 2006; Moller et al., 1971; Ormeño et al., 1999; Sakuma & Kida, 2010; Shiino et al., 2016). The suprahyoid muscles initiate hyoid bone excursion during swallowing, while the infrahyoid muscles induce superior laryngeal elevation. Given that the electromyographic activities of the suprahyoid muscles coincide with the initiation of swallowing, analyzing these activities yields insights into the onset and duration of pharyngeal swallowing. Consequently, extensive research has been conducted to elucidate the effects of posture on suprahyoid electromyographic activities during swallowing. Despite these efforts, a consensus on the influence of posture on suprahyoid muscle activity during swallowing remains elusive, with divergent findings across studies.

Lund et al. (1970) demonstrated that electromyographic activity in the digastric muscle was more pronounced in the upright position than in the supine position during saliva swallowing. Shiino et al. (2016) reported posture‐dependent significant differences in burst duration and falling time of suprahyoid electromyography during involuntary swallowing of orally infused water. Ayuse et al. (2006) observed a significantly prolonged duration of swallowing apnea and submental electromyography burst in the 60‐degree reclining with a 60‐degree chin‐tuck position, in comparison to both upright sitting and 30‐degree reclining positions. In contrast, Ormeño et al. (1999) contended that body posture exerted no discernible effect on integrated electromyographic activity during saliva swallowing. A series of studies by Inagaki and colleagues (Inagaki et al., 2007, 2008, 2009b) posited that alterations in body position failed to elicit changes in suprahyoid electromyographic activity. Sakuma and Kida's findings suggested that the group‐averaged duration and amplitude of suprahyoid activity while swallowing 10 g of jelly did not exhibit significant variations with alterations in body angle (Sakuma & Kida, 2010). Previous studies have been conducted by swallowing food boluses of various quantities and properties. Since the ingested food bolus stimulates various sensory receptors in the oropharyngeal area and modulates swallowing, the discrepancy of these studies may be due to the sensory stimulation of the oropharyngeal area caused by the differences in the food bolus used.

Voluntary swallowing is instigated by the convergence of central and peripheral inputs into the swallowing central pattern generator (CPG) located in the medulla oblongata (Miller, 2008). Consequently, sensory inputs from a food bolus play a pivotal role in influencing swallowing, with sensory input from the pharyngeal region specifically modulating the process (Steele & Miller, 2010). The effect of sensory stimulation on swallowing has been studied from the perspective of dysphagia rehabilitation. It has been confirmed that sensory effects of water (Kitada et al., 2010; Nakamura et al., 2013; Yahagi et al., 2008), cold mechanical stimulation (Rosenbek et al., 1991, 1998), bolus volume or viscosity modification (Dantas et al., 1990; Perlman et al., 1993), and chemical stimulation including taste and carbonation (Leow et al., 2007; Tsuchiya et al., 2023) influence swallowing in humans.

Application of water to the larynx induces the swallowing reflex through the stimulation of water‐sensitive fibers in the superior laryngeal nerve, a branch of the vagus nerve (Shingai, 1979). In rabbits, the activation of these water‐sensitive fibers within the superior laryngeal nerve has been demonstrated to be suppressed by the application of hypertonic NaCl solution (Shingai & Shimada, 1976). Yahagi et al. (2008) investigated the effect of water and NaCl solution application on voluntary swallowing in humans by administering these solutions to the pharyngeal region. To minimize mechanoreceptor stimulation, the researchers used an orally inserted fine tube to deliver the solutions at a slow infusion rate (0.2 mL/min). Their findings revealed that water application reduced swallowing intervals (SIs) during repetitive voluntary swallowing compared to NaCl solution application. These results imply the existence of water‐sensitive nerve fibers in the human pharynx and suggest that NaCl inhibits the excitation of these fibers in humans. Many of the previous studies that evaluated suprahyoid muscle activity to determine the effect of positional changes on swallowing assessed relatively large volumes of water swallowing. Application of large amounts of water induces excitation of water‐sensing neurons and mechanoreceptors that are located in the pharyngeal mucosa. Therefore, the effect of pharyngeal stimulation by applying large amounts of water on swallowing during positional changes cannot be ignored. The facilitative effect of sensory information from the pharyngeal region on swallowing may obscure the impact of changes in body position on the process.

To elucidate the influence of postural change on swallowing, it becomes imperative to mitigate the influence of oropharyngeal sensory stimulation on the induction of swallowing as much as possible. The low‐velocity injection of a 0.3 M NaCl solution into the pharynx, devoid of sensory stimulation to trigger swallowing, would unveil the effect of postural changes on swallowing in the absence of sensory induction. Consequently, our hypothesis posits that changes in posture impact suprahyoid electromyography activities and, consequently, repetitive swallowing initiation. This study aims to investigate the effect of postural changes on repetitive voluntary swallowing by monitoring suprahyoid electromyography during the administration of NaCl solution to the pharyngeal region. In other words, our hypothesis is that body reclining affects the ease of performing voluntary swallowing and conceptually prolongs swallowing intervals during voluntary swallowing.

## 2 MATERIALS AND METHODS

Fourteen healthy female volunteers (mean age, 30.27 years; range, 21–60 years) with no oropharyngeal disorders, not taking any medication, and without impairments in taste or olfaction were enrolled in this study. All participants were recruited from university staff and students and enrolled by researchers. Subjects were instructed to fast and abstain from drinking for at least 1 hour prior to the experiment. The experiment was conducted in the laboratory of the Department of Oral Health Sciences. The minimum required sample size was calculated using G*Power software version 3.1.9.7 (Heinrich Heine University, Düsseldorf, Germany). With an effect size of 0.5, an α error probability of 0.05, and a power of 85%, the calculation yielded a required total number of 14 participants. All participants provided written informed consent prior to their inclusion in the study. The study protocol was approved by the Ethics Committee of Baika Women's University (approval number: [2021–0198]). All procedures were conducted in accordance with the ethical standards of the institutional research committee and with the 1964 Helsinki Declaration and its later amendments.

**FIGURE 1:**
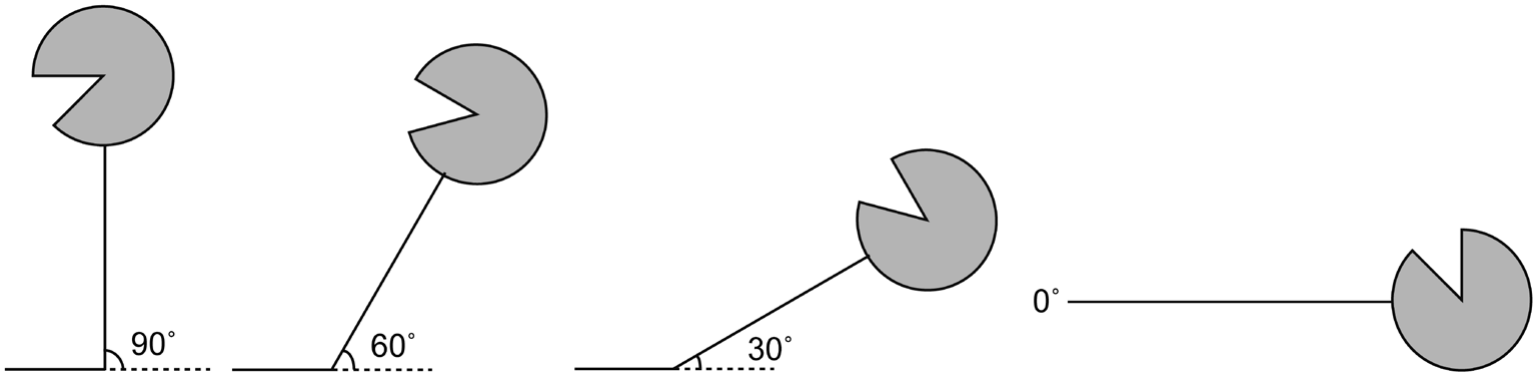
The four different postures used in this study. Postures were as upright (90°), 60° reclining (60°), 30° reclining (30°), and supine (0°). In the upright position, the subject's Frankfort plane was set parallel to the floor.

Subjects sat in a reclining dental chair, and bipolar surface electrodes were placed on their right suprahyoid muscles. The chair was reclined to body angles of 90°, 60°, and 30° from the floor, and the subjects lay supine on the floor for a body angle of 0° (Figure 1). 0.3 M NaCl solution was applied to the pharyngeal region through a fine silicone tube of 1‐mm outer diameter (94–0451‐4; Sansyo, Tokyo, Japan) that was orally inserted into the throat. We chose 0.3 M NaCl solution because of its inhibitory effects on the excitation of water‐sensitive fibers. Solutions were used at room temperature (24°C). The tip of the tube was positioned at a distance of 12 cm from the mandibular incisors; this distance corresponds to the position of the pharyngeal region. Infusion of a small amount of solution into the tube at this tip position did not give rise to any taste, burning, stinging, tingling, numbness, or tactile sensation. To minimize the mechanical effect of infusion, solutions were delivered through the tube using an infusion pump (SP100i; World Precision Instruments, Sarasota, FL, USA) at a very slow infusion rate (0.2 mL/min). The order of the reclining position was randomized by using dice and random numbers. Participants were instructed to perform repetitive swallowing as rapidly as possible after the onset of infusion. During each infusion, repetitive swallowing was performed for about 3 minutes. Between trials, the participants were told to drink water as they wished, and the next swallowing session was performed after an interval of 7 minutes. Surface EMG was recorded from the left suprahyoid muscles to examine muscle activity associated with swallowing. Two silver/silver chloride electrodes were taped under the chin, and surface EMG was recorded with a biological signal acquisition system (PowerLab 26 T; ADInstruments, Colorado Springs, CO). Signals from the EMG electrodes were amplified and filtered (low‐pass, 30 Hz; high‐pass, 2 kHz), and the amplified EMG signals were stored on a personal computer. The sampling rate was 20 kHz. Data analysis was performed using the PowerLab software package (LabChart ver. 8.1.16; ADInstruments, Colorado Springs, CO). Participants pressed a button connected to the PowerLab system immediately after swallowing to record the actual swallowing time.

The swallowing interval (SI) was measured between two consecutive marks indicated by pressing the button. EMG burst activity confirmed that the participant was actually swallowing when the button was pressed. The first five SIs were discarded due to the potential influence of residual saliva. The average of the next five consecutive SIs was taken as the participant's SI. To calculate the duration and maximum amplitude of EMG activity during swallowing, EMG signals were integrated with a time constant of 0.05 s. The integrated EMGs recorded at rest were rectified for 5 s, and the mean value ±SD was obtained as a control. In this study, the onset of swallowing was defined as the point at which the integrated EMG signal activity exceeded 2 SD from the mean values at rest, leading to the swallowing event. The offset of swallowing was defined as the point at which the signal activity fell below 2 SD from the mean values at rest. To calculate the duration of suprahyoid muscle activity, the time of offset was subtracted from the time of onset. The amplitude of suprahyoid muscle activity was evaluated for the defined duration described above. To assess swallowing performance, the amplitude of muscle activity was calculated as the difference between baseline and swallowing for the defined duration. To account for variations in integrated EMG signals, the maximum amplitude and duration of integrated EMG activity in the suprahyoid muscle group during swallowing were calculated by averaging the first five of six consecutive swallowing muscle bursts used to measure the SIs.

Statistical analysis was conducted using KaleidaGraph version 4.5 (Synergy Software, Essex, VT). The mean values of SI and EMG data for each individual were compared across different body postures using one‐way repeated‐measures analysis of variance (ANOVA), followed by Tukey's honest significant difference (HSD) post‐hoc test for multiple comparisons. The level of significance was set at *p* < 0.05. Values are presented as mean ± standard error of the mean.

## 3 RESULTS

**FIGURE 2:**
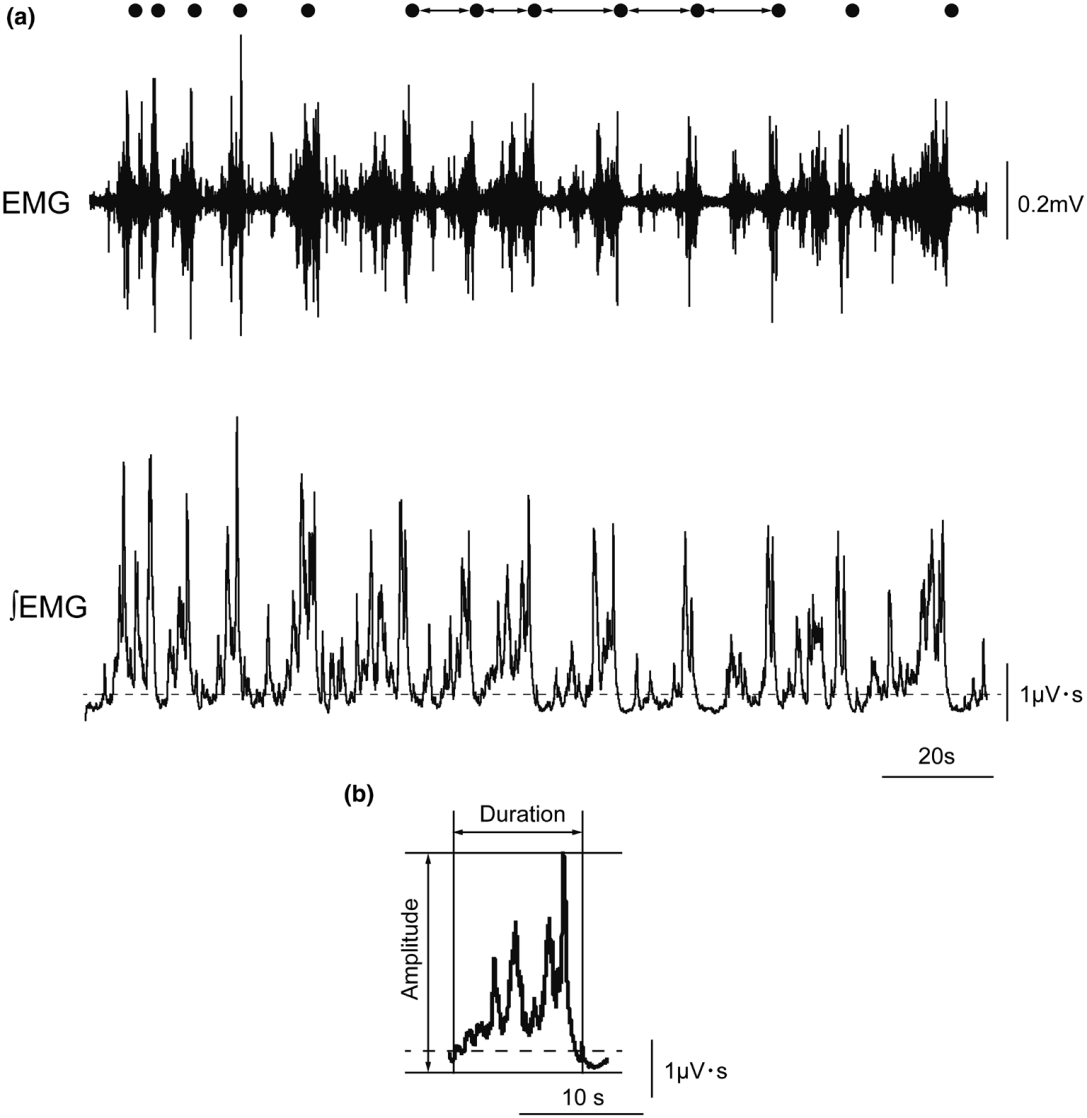
Example of recordings during swallowing of 0.3 M NaCl solution. (a) Raw and integrated data of EMG recordings of suprahyoid muscles. The dots above raw EMG recording indicate the actual swallowing time points. To reduce the influence of residual saliva, the SI was obtained by an average of five continuous SIs (1–5). The first five SIs after the beginning of swallowing were discarded to reduce the influence of residual saliva on the SI. The dotted line in integrated EMG indicates the EMG signal activity of 2 SD from the mean values at rest. (b) Integrated data of EMG recording of suprahyoid muscles in one swallow. The duration was defined as the time between the onset and offset of the burst. Amplitude was defined as the peak value from the resting level of EMG for the duration.

**FIGURE 3:**
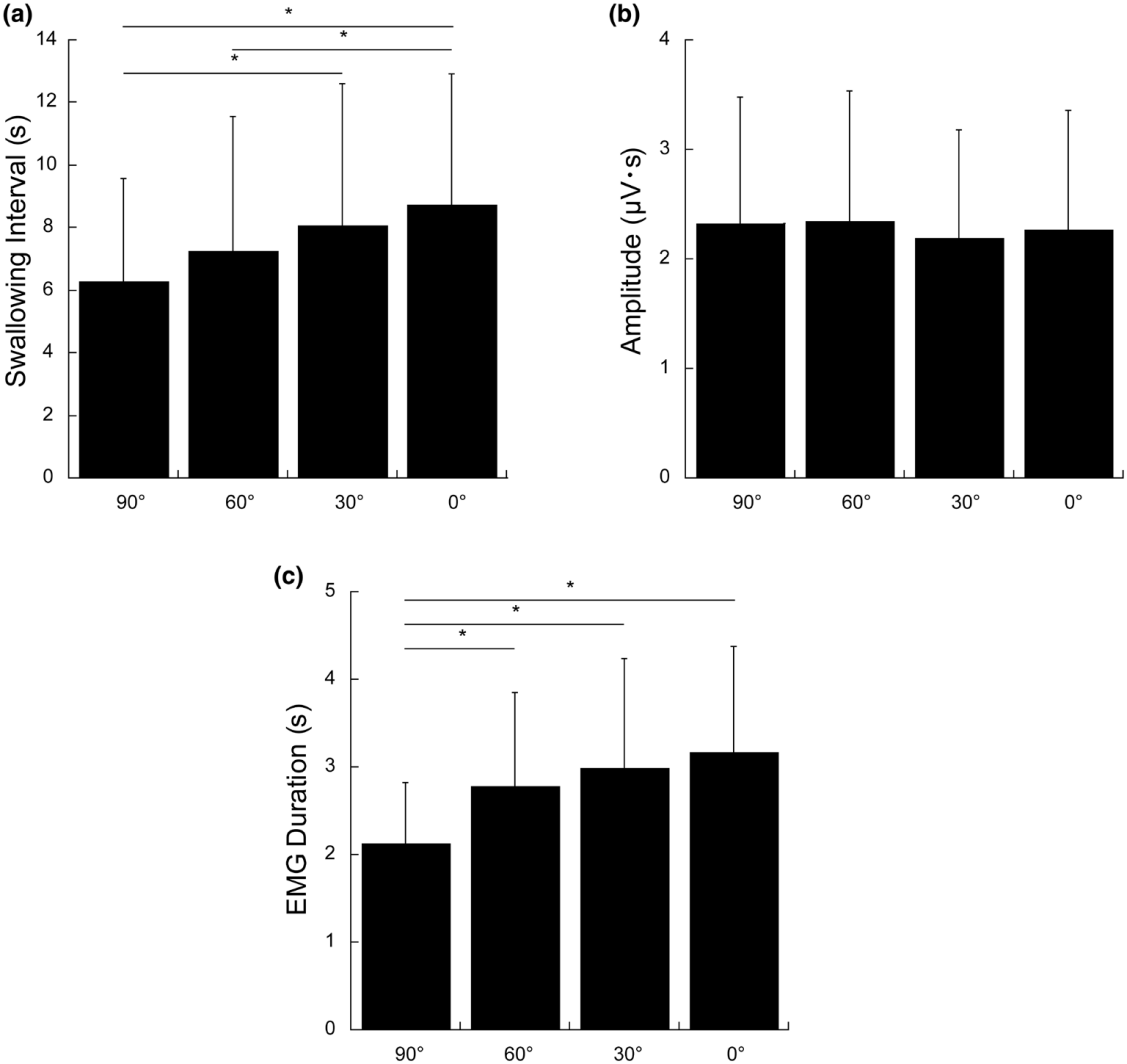
Effects of body angulation on the SI (a), EMG amplitude (b), and EMG burst duration (c). SI was 6.25 ± 3.32 s for 90, 7.24 ± 4.3 s for 60, 8.06 ± 4.54 s for 30, and 8.7 ± 4.23 s for 0. There was a significant difference between 90 and 30, and 90 and 0, 60 and 0. Peak amplitude was 2.33 ± 1.15 μV･s for 90, 2.34 ± 1.19 μV･s for 60, 2.19 ± 0.99 μV･s for 30, and 2.26 ± 1.1 μV･s for 0. There were no differences between the postures. Burst duration was 2.13 ± 0.69 s for 90, 2.78 ± 1.07 s for 60, 2.98 ± 1.25 s for 30, and 3.17 ± 1.2 s for 0. There was a significant difference between 90 and 60, 90 and 30, and 90 and 0. *p < 0.05.

Data obtained from all subjects were analyzed. Figure 2 shows typical EMG recordings of the suprahyoid muscles and actual swallowing points during repeated voluntary swallowing at the maximum frequency with an infusion of the solution into the pharyngeal region at a low infusion rate. Figure 3 shows the group averages of the SI, duration, and amplitude of suprahyoid muscle activity in the four different body angles for swallowing. Effect of body position on EMG burst in the pharyngeal 0.3 M NaCl infusion. SI was 6.25 ± 3.32 s for 90, 7.24 ± 4.3 s for 60, 8.06 ± 4.54 s for 30, and 8.7 ± 4.23 s for 0. Peak amplitude was 2.33 ± 1.15 μV･s for 90, 2.34 ± 1.19 μV･s for 60, 2.19 ± 0.99 μV･s for 30, and 2.26 ± 1.1 μV･s for 0. Burst duration was 2.13 ± 0.69 s for 90, 2.78 ± 1.07 s for 60, 2.98 ± 1.25 s for 30, and 3.17 ± 1.2 s for 0. The amplitude of the EMG did not indicate significant differences among each angle. The SI and EMG duration were significantly affected depending on the body angle. The upright position indicated a significantly shorter SI than the 60° reclining position or the supine position (0° recline). Furthermore, the 30° reclining position indicated a significantly shorter swallowing interval than the supine position. The upright position indicated a significantly shorter EMG duration than the 60°, 30°, and 0° supine positions.

## 4 DISCUSSION

This study investigated the effects of body reclining on swallowing in the absence of sensory information from the pharyngeal region. Our findings demonstrate a tilt‐dependent increase in swallowing intervals and the duration of suprahyoid electromyography bursts during repetitive voluntary swallowing. Specifically, a significant increase in electromyography burst duration and swallowing intervals was observed in the supine (0°) position, as compared to the upright (90°) and 60° positions.

The contraction of the suprahyoid muscles, responsible for hyoid bone elevation and laryngeal ascent during the pharyngeal phase of swallowing, assumes a pivotal role in preventing the entry of the food bolus into the trachea (Crary Ma et al., 2006). However, investigations into the effects of body position during swallowing on suprahyoid muscle activity have yielded inconclusive results. While several studies have indicated that alterations in body angulation do not yield significant effects on suprahyoid electromyography activities (Inagaki et al., 2008, 2009a, 2009b; Ormeño et al., 1999; Sakuma & Kida, 2010), our study demonstrates a prolonged suprahyoid electromyography duration with changes in body angulation. This discrepancy may be attributed to variations in the nature of the bolus swallowed, as peripheral sensation plays a crucial role in swallowing induction. Ferris et al. (2021) observed increased contractility of the pharyngeal region in association with increased food bolus volume, suggesting that activation of neural circuits by excitation of mechanoreceptors in the pharyngeal region may also be involved during voluntary swallowing. Yahagi et al. (2008) indicated that water application shortened the swallowing interval (SI) during repetitive voluntary swallowing compared with NaCl solution application. They concluded that water‐sensitive nerve fibers are present in the human pharynx and that applying NaCl solution inhibits the excitation of water‐sensitive nerve fibers. Stimulation by a large volume of water bolus in the pharyngeal region is a powerful swallow‐evoking stimulus, which may have masked the changes in electromyographic duration of the suprahyoid muscle group due to the change in body position. In our research, the slow infusion of 0.3 M NaCl into the pharyngeal region minimizes activation of water‐sensitive fibers and mechanoreceptors in the pharyngeal region, revealing the apparent effect of postural change on suprahyoid electromyographic activity. This is supported by the results of other studies that have eliminated the influence of sensation in swallowing. Similar to our results, Shiino et al. (2016) showed that the supine position prolongs the duration of EMG bursts. They recorded the muscle activity during swallowing when a small amount of water (0.3 mL/min) was injected into the posterior of the tongue. As with the slow injection of the NaCl solution used in our study into the pharynx, it has been shown that slow injection of water into the posterior of the tongue does not induce swallowing (Yahagi et al., 2008).

Interestingly, consistent with previous studies, the maximum amplitude of suprahyoid muscle EMG was not affected by reclining position (Inagaki et al., 2007, 2008, 2009b; Ormeño et al., 1999). Training of the suprahyoid muscles has been investigated in both healthy individuals and patients with dysphagia for use in rehabilitation of patients with dysphagia. Indeed, it has been reported that training increases the maximum amplitude of suprahyoid muscle EMG in both healthy individuals and patients with dysphagia (Chang et al., 2021; Cho et al., 2023; Kılınç et al., 2020; Park et al., 2020). However, the training effects on suprahyoid muscle activity during actual swallowing have primarily been reported in healthy elderly individuals (Cho et al., 2023). Since the majority of subjects in the present study were relatively young, it is possible that reclining did not affect the maximum amplitude of suprahyoid muscle electromyography. Given that prolonged muscle activity was observed, we considered that in young healthy individuals, the suprahyoid muscles maintain swallowing function by adjusting their activity patterns rather than amplitude in response to postural changes. While EMG amplitude remained unchanged, the significant prolongation of muscle activation duration represents a different but equally important aspect of neuromuscular adaptation. This temporal modulation may be therapeutically relevant for dysphagia rehabilitation, as sustained muscle activation could contribute to improved swallowing safety and efficiency through mechanisms distinct from peak force generation. Future studies should investigate the effects of reclining on suprahyoid muscle activity in healthy elderly individuals and patients with dysphagia.

Notably, our study is the first to report that changes in body angle during repetitive swallowing influence swallowing intervals. A significant prolongation of swallowing intervals was noted in the supine and 30° reclined positions, compared to the upright position. These results suggest that performing repetitive voluntary swallowing is more effortful in positions reclining beyond 30°. Several studies revealed that slower application of relatively high concentrations of NaCl solution into the pharyngeal region was not an effective stimulus for inducing swallowing. Kitada et al. (Kitada et al., 2010) reported that in situations where the input to the swallowing CPG from the periphery is reduced, the input from the cerebral cortex becomes dominant. The potential influence of posture on brain function is a plausible explanation, as our study, conducted under conditions of low sensory impact, primarily relies on central inputs for swallowing initiation. Previous research has indicated that body posture affects brain activities, with upright positions inducing increased β and γ frequency oscillations in the cerebral cortex compared to supine postures. In particular, high γ oscillations in the upright position indicated greater activities than the supine position in the precentral gyrus, which is the motor area (Lifshitz et al., 2017). Hashimoto et al. (2021) recorded EEGs during swallowing in epilepsy patients with intracranial electrodes in place. They reported that the peak of high γ power appeared in cortical areas associated with swallowing during voluntary swallowing phases. Consequently, our findings may reflect a reduction in cortical swallowing‐related motor area activity attributed to body reclining.

Previous studies have reported a relationship between the degree of reclining and the subjective evaluation of the ease of swallowing the test food. Sakuma and Kida (2010) reported that the evaluation score for ease of swallowing at a 60° reclining position (equivalent to a 30° reclining position in this study) was high, but there was no significant difference in the group mean between the body position angles. On the other hand, in a study by Lu et al. (2023) when the ease of swallowing was evaluated on a 5‐point subjective scale, it was reported that the degree of difficulty in swallowing increased significantly when the angle of reclining was 60 degrees and the subject was lying on their back, compared to when the angle of reclining was 30 degrees or the subject was upright. These results are consistent with the shortening of SI observed in the 30‐degree and upright positions in this study. The prolongation of the SI during repetitive voluntary swallowing in the reclining position shown in this study is thought to indicate that body reclining at an angle of 60 degrees or more made swallowing more difficult. We believe that the results of this study support the results presented by Lu et al. (2023).

The biomechanical role of gravity in swallowing physiology presents a complex and nuanced picture that challenges simplistic assumptions about gravitational assistance. While established physiological principles confirm that gravity assists esophageal peristalsis in the upright position through coordinated relaxation and propulsive waves, direct experimental evidence reveals significant anatomical specificity in gravitational effects (Matsuo & Palmer, 2008). Previous studies have demonstrated that the notable change observed during the transition from the upright to the supine position is the pressure change in the pharynx and esophagus during swallowing. Johnsson et al. (1995) conducted the seminal investigation showing that while gravity does not significantly influence pharyngeal bolus transport velocity or transit times, important compensatory biomechanical adaptations occur, with hypopharyngeal intrabolus pressure increasing significantly in horizontal positions compared with upright, resulting in increased maximal sphincter diameters and shorter duration of sphincter opening. Similarly, Rosen et al. (2018) reported increased pharyngeal pressure and pharyngeal‐upper esophageal sphincter pressure gradients when participants swallowed in supine positions, representing neurophysiological adaptations to gravitational changes. Our current EMG findings may provide complementary evidence for these position‐dependent adaptations, demonstrating that while suprahyoid muscle amplitude remains unchanged across body positions, EMG duration significantly increases in horizontal positioning. This temporal compensation pattern may explain how unchanged muscle activation intensity contributes to the increased pharyngeal pressures documented manometrically, suggesting that the neuromuscular system maintains effective swallowing through temporal rather than amplitude‐based adaptations. The formation of pharyngeal pressure during swallowing results from coordinated activity of multiple muscle groups beyond the suprahyoid muscles, including pharyngeal constrictors and soft palate muscles; therefore, the prolonged suprahyoid muscle contraction observed in our study may contribute to cumulative force‐time effects that manifest as pressure changes rather than directly modulating primary pressure generation mechanisms.

This study found that when the reclining angle is 30 degrees or more, the interval between swallows becomes longer, making it challenging to perform voluntary, continuous swallowing. It has been reported that body reclining can reduce the amount of food residue in the pharynx. Park et al. (2013) showed that food residue in the epiglottal fossa decreased, but that in the piriform fossa increased when the upright and 45‐degree reclining positions were compared. In the study by Benjapornlert et al. (2020) the upright and 30‐ and 45‐degree reclining positions from upright were compared and showed that both the piriform fossa and the epiglottal fossa had less residue in the reclining posture. Both studies pointed out that in the reclining posture, the food bolus is more likely to move along the posterior wall of the pharynx due to the effects of gravity and to pass through the UES, and that this reduces the risk of penetration into the larynx and aspiration. In other words, while upright or reclining positions of up to 30 degrees make it easier to perform swallowing than 60‐degree reclining or supine positions, upright or reclining positions of up to 30 degrees increase the risk of aspiration because the amount of food residue in the pharynx increases compared to 45‐degree reclining positions. Therefore, when applying the reclining position to patients with dysphagia, it is necessary to strike a balance between the ease of swallowing and the possibility of food residue in the pharynx. In this study, a 30‐degree body recline significantly shortened the time between swallows compared to a 60‐degree or supine position. Although we did not investigate a 45‐degree recline, it is between 30 and 60 degrees, which showed a significant difference, so we think applying a 45‐degree recline to patients with dysphagia is reasonable.

Several limitations must be considered in this study. First, limiting the subjects to women only may restrict the generalizability of the findings obtained. It is known that there are gender differences in the function of swallowing and the structure of organs related to swallowing (Dantas et al., 2009; Van Herwaarden et al., 2003). Especially, the hyoid bone shifts when the body is laid flat, and Perry et al. (Perry et al., 2012) also reported gender differences in the position of the hyoid bone in the prone and upright postures. Because the position of the hyoid bone may affect the EMG activity of the suprahyoid muscles, this research was conducted with female subjects only. Future experiments should include male subjects to gain a more comprehensive understanding of the effects of postural changes on swallowing. Secondly, our measurements focused primarily on suprahyoid muscle activity and swallowing intervals. Additional physiological parameters such as hyoid bone movement, upper esophageal sphincter opening, laryngeal elevation, and pharyngeal residue were not assessed. Incorporating these measurements through videofluoroscopy, manometer or endoscopy would provide a more comprehensive understanding of the biomechanical effects of body inclination on swallowing. Thirdly, this study is the absence of direct measurements of brain function related to swallowing. In the discussion section, we attempted to interpret the effects of body position changes on swallowing intervals and suprahyoid EMG burst duration from the perspective of altered brain function, but these interpretations are speculative and based on previous research (Hashimoto et al., 2021; Lifshitz et al., 2017). To directly verify how positional change affect swallowing‐related brain activity, additional studies using neurophysiological methods such as electroencephalography, functional magnetic resonance imaging, or transcranial magnetic stimulation would be necessary. Future research directly measuring swallowing‐related brain activity during different body positions would provide clearer insights into the neural mechanisms underlying the behavioral changes observed in our study.

In summary, this study revealed a posture tilt‐dependent prolongation of suprahyoid electromyography burst duration and swallowing intervals during the swallowing of 0.3 M NaCl injected into the pharyngeal region. The supine position or a 30‐degree elevated position from it may impede the induction of swallowing, elevating the risk of aspiration. Consequently, feeding and swallowing training for dysphagia patients is recommended in more than a 30‐degree elevated position from the supine position to optimize rehabilitation outcomes.

## AUTHOR CONTRIBUTIONS

Conceptualization: Yumiko Kubo, Hideyuki Fukami. Data curation: Yumiko Kubo, Hideyuki Fukami. Formal analysis: Yumiko Kubo. Investigation: Yumiko Kubo. Funding acquisition: Yumiko Kubo, Hideyuki Fukami. Methodology: Hideyuki Fukami. Project administration: Hideyuki Fukami. Resources: Yumiko Kubo, Hideyuki Fukami. Supervision: Hideyuki Fukami. Validation: Yumiko Kubo, Hideyuki Fukami. Writing—original draft: Yumiko Kubo, Hideyuki Fukami.

## CONFLICT OF INTEREST STATEMENT

The authors declare that the research was conducted in the absence of any commercial or financial relationships that could be construed as a potential conflict of interest.

## ETHICS STATEMENT

Ethics approval was received from the Ethics Committee of Baika Women’s University (approval number: 2021‐0198).

## Data Availability

The datasets generated during and/or analyzed during the current study are available from the corresponding author on reasonable request.
